# Wavefront shaping with nonlinear four-wave mixing

**DOI:** 10.1038/s41598-023-29621-w

**Published:** 2023-02-16

**Authors:** Dongyi Shen, Jianjun Cao, Wenjie Wan

**Affiliations:** 1 State Key Laboratory of Advanced Optical Communication Systems and Networks, School of Physics and Astronomy, Shanghai Jiao Tong University, Shanghai, 200240 China; 2 School of Science, Jiangnan University, Wuxi, 214122 China; 3 University of Michigan-Shanghai Jiao Tong University Joint Institute, Shanghai Jiao Tong University, Shanghai, 200240 China

**Keywords:** Nonlinear optics, Metamaterials

## Abstract

Wavefront manipulations have enabled wide applications across many interdisciplinary fields ranging from optics and microwaves to acoustics. However, the realizations of such functional surfaces heavily rely on micro/nanofabrication to define the structured surfaces, which are fixed and only work within a limited spectrum. To address these issues, previous attempts combining tunable materials like liquid crystal or phase-change ones onto the metasurfaces have permitted extra tunability and working spectra, however, these additional layers bring in inevitable loss and complicate the fabrication. Here we demonstrate a fabrication-free tunable flat slab using a nonlinear four-wave mixing process. By wavefront-shaping the pump onto the flat slab, we can successfully tune the effective nonlinear refraction angle of the emitting FWM beams according to the phase-matching condition. In this manner, a focusing and a defocusing nonlinear of FWM beam through the flat slab have been demonstrated with a converging and a diverging pump wavefronts, respectively. Furthermore, a beam steering scheme over a 20° angle has been realized through a non-degenerate four-wave mixing process by introducing a second pump. These features open up a door to manipulating light propagation in an all-optical manner, paving the way to more functional and tunable flat slab devices in the applications of imaging and all-optical information.

## Introduction

Wavefront manipulation through metasurfaces becomes an emerging field with many promising applications. Metasurfaces, two-dimensional thin films constructed by subwavelength phase-disturbing elements, have exhibited enormous capabilities in molding flow of wave propagation in optics, acoustic and microwave^1–9^, achieving functional applications in lenses, polarization controlling, holograms, and beam-shaping^10–19^. Such thin-film structures can be considered as variations of metamaterials with a reduced dimension from 3 to 2D, this greatly eases the rigid fabrication requirements and makes ultra-flat functional optical components comparable to or even exceeding conventional optics. Unlike traditional optical elements such as lenses which rely on the spatially varying indexes to modify propagating lights’ wavefronts to achieve focusing, metasurfaces rely on collective interferences from a cluster of locally defined subwavelength scatters, i.e., optical antennas, to shape wavefronts, polarization, refraction and reflection properties^20–22^. This process can be traced back to the example of Fresnel zone plates^23,24^ in conventional optics, but metasurfaces allow much finer structuring of local subwavelength scatters, eliminating higher order diffraction, hence better efficiency can be obtained.

However, such flat metasurfaces are usually constructed through micro/nanofabrication with metallic and dielectric materials^1,3,25^, where the structures are *non-tunable* due to the fixed design. Furthermore, their functionalities would suffer from material absorption and scattering loss. Although prior works have demonstrated metal-free metasurfaces based on high-index dielectric materials^26^, and tunability of metasurfaces can be achieved by combining external liquid crystal or phase-change materials^27–29^, it still remains a challenging task to fabricate and integrate these micro/nanostructures. Alternatively, nonlinear metasurfaces were implemented by introducing nonlinearity to tackle the problem of tunability, e.g., nonlinear plasmonic scatters^30,31^; moreover, negative refraction based on nonlinear wave mixing has been demonstrated in a metal thin film^32^ and a flat dielectric slab for imaging applications^33–35^. These nonlinear approaches open up a new avenue to study functional metasurfaces on a 2D geometry in a tunable manner.

In this work, we experimentally demonstrate a fabrication-free tunable flat slab using nonlinear four-wave mixing (FWM) process by wavefront-shaped pump beams. Such an FWM process involves frequency up-conversion and possesses diverse propagation features following phase matching conditions, which provides tools for wavefront manipulations. Previously, nonlinear effective negative refractions (NR) have been realized under nonlinear χ^(3)^ and χ^(2)^ susceptibility^33–35^. However, such nonlinear refractive relations are fixed and not tunable either, attributing to the rigid phase matching conditions between the incident signal and the refracted beam. Alternatively, in the current work, wavefront shaping is implemented onto the pump beams instead of the signals in prior works^33–35^, such that a tunable and controllable FWM beam can be obtained. Accordingly, we show spatially-varying refraction relations (pump to FWM beam) via an FWM process in a χ^(3)^ glass slab; combined with a wavefront shaped pump, a focusing and defocusing FWM are demonstrated with a converging and a diverging pump. Furthermore, to gain a more tunable angle in the beam steering application, we introduce an additional degree of tunability by exploring a non-degenerate FWM, which broadens the steering angle up to over 20 degrees, much larger than prior works^33–35^. These features may pave a new way to more functional and tunable flat slab devices in the applications of imaging and all-optical information.

## Principle and method

**Figure 1:**
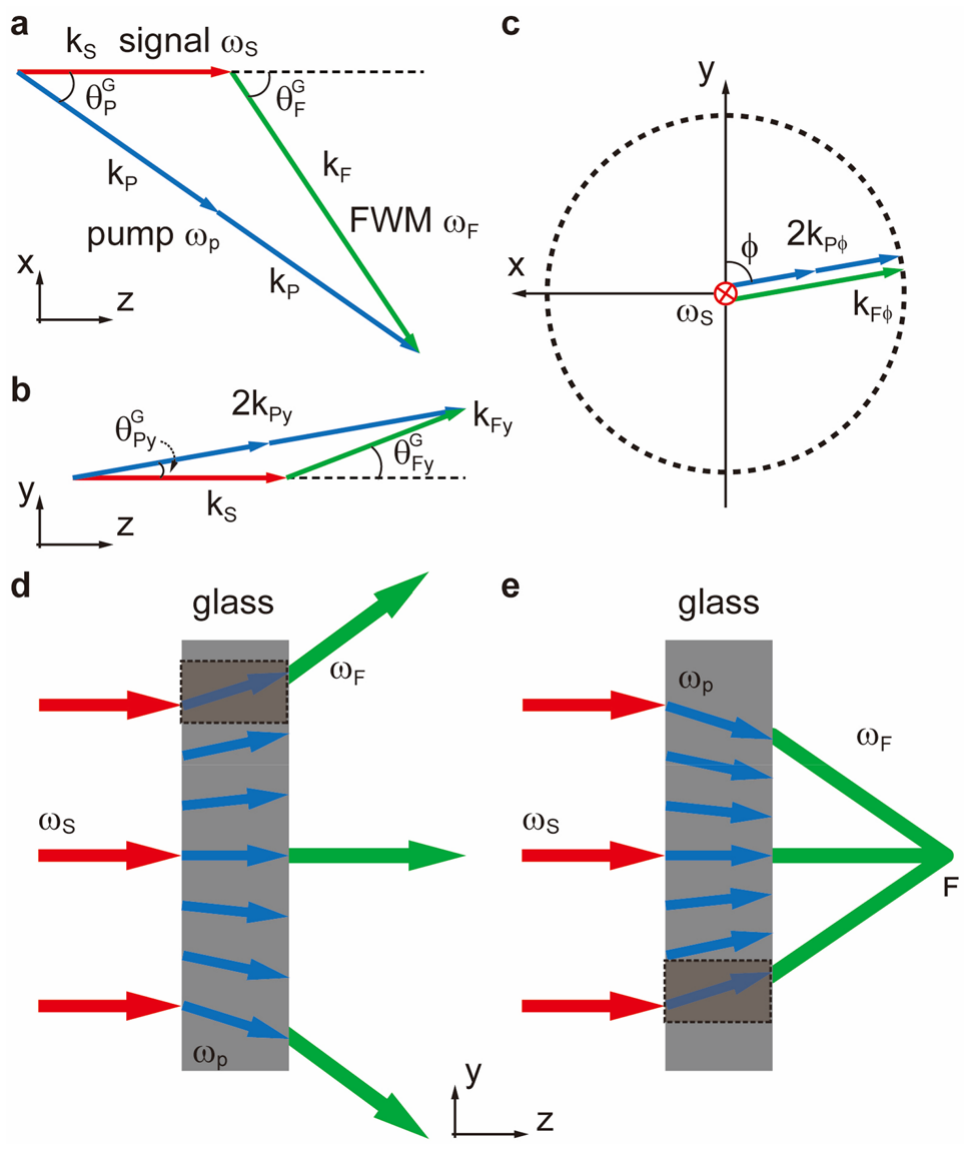
Schematic of nonlinear refractions of a degenerate FWM. Phase matching triangle in the *x* ~ *z*, *y* ~ *z,* and *x* ~ *y* planes, *k* symbolizes the wavevectors for each component in the FWM process. (**a**) In the *x* ~ *z* plane, ${\theta }_{p}^{G}$ and ${\theta }_{F}^{G}$ are the incident and emittance angles within the glass slab fulfilling the phase matching condition $2\vec{k}_{p} \, - \,\vec{k}_{s} \, = \,\vec{k}_{_F}$. (**b**) The matching condition of the wavevectors in the *y* ~ *z* plane, marked as 2*k*_*py*_ and *k*_*Fy*_ form a new triangle-like relation with unchanged *k*_*s*_ in direction + *z*, where a new pair of angular relation of ${\theta }_{py}^{G}$ and ${\theta }_{Fy}^{G}$ is brought in. Such a relation could be tuned within tolerance. (**c**) The projections of the above wavevectors in the *x* ~ *y* plane denoting as *k*_*pϕ*_ and *k*_*Fϕ*_ are a few degrees deviated from the orthogonal (*ϕ* = 90°) position. All the possible endpoints for *2k*_*pϕ*_ and *k*_*Fϕ*_ lead to a dashed circle in the *x* ~ *y* plane. (**d**) For the case of a diverging pump beam onto the slab, the FWM beam also exhibits a diverging trend accordingly, due to the local nonlinear refraction relations locally defined in the (**c**). (**e**) A focusing pump onto the slab causes a focusing effect on the FWM beam instead.

In our proposed framework as shown in Fig. 1, the degenerate nonlinear four-wave mixing (FWM) process occurs within a BK-7 glass slab of 1 mm thickness via third-order nonlinearity susceptibility χ^(3)^. Here we can obtain the phase matching conditions and energy conservation relations as: $2\vec{k}_{p} \, - \,\vec{k}_{s} \, = \,\vec{k}_{_F}$ (Fig. 1a,b), and $2{\omega }_{p}- {\omega }_{s}= {\omega } _{_F}$, where the subscripts of each *k* and $\omega$ symbolize the wavevectors and frequencies for degenerated pumps, signal, and FWM beam respectively. To mimic the refraction process, in our case, the signal serves as the incident beam, and the FWM beam serves as the refracted beam out of the nonlinear glass slab^32–35^. In this manner, extra tunability can be gained by the wavefront-shaped pump beam to define the spatial-dependent phase change in the “flat slab”, enabling controllable nonlinear refraction. Experimentally, the pumps are from a Ti: Sapphire femtosecond laser with a pulse duration of ~ 75 fs, centered wavelength at *λ*_*p*_ = 800 nm. Along with the pumps, there derives the signal centered at *λ*_*s*_ = 1300 nm through an optical parametric amplifier (OPA). The resultant FWM beam is thus to be *λ*_*F*_ = 578 nm in wavelength. The pump and signal beam share the same repetition rate of 1 kHz. Since these are all ultrafast pulses, the actual linewidth for the pump beam is about 50 nm and about 100 nm for the signal beam.

To understand the working principles of the nonlinear flat slab, the phase matching condition is the key. Here the phase matching conditions are fulfilled under the circumstance that the signal is arranged normally incident into the glass slab (Fig. 1). The exact angles for the incident and emittance at the boundary of the slab can be estimated by phase matching relation considering refraction index of the glass. For simplicity, we derive the phase matching condition in directions *z* and *x* separately as:

1 $$k_{s} = 2k_{p} \cos \theta_{p}^{G} - k_{_F} \cos \theta_{F}^{G} .$$

2 $$2k_{p} \sin \theta_{p}^{G} = k_{_F} \sin \theta_{F}^{G} .$$

Here, *k*_*s*_, *k*_*p,*_ and *k*_*F*_ are the wavevectors of the participant beams inside the glass slab. ${\theta }_{p}^{G}$ and ${\theta }_{F}^{G}$ also present the phase-matching angles inside the glass slab(Fig. 1a). Moreover, the angles outside the glass slab (*θ*_*p*_, *θ*_*F*_) are denoted as:

3 $$n_{p} \sin \theta_{p}^{G} = \sin \theta_{p} ,$$

4 $$n_{_F} \sin \theta_{F}^{G} = \sin \theta_{F},$$

where *n*_*p*_ and *n*_*F*_ are the refractive indexes of the BK-7 glass for the pump and the FWM beam. For the wavelengths studied in this scheme, the angles outside the slab can be calculated as *θ*_*p*_ = 6.44º for the pump, and *θ*_*F*_ = 9.34º for the FWM beam.

In Fig. 1a, the participant wavevectors *k*_*s*_, *k*_*p*_ and *k*_*F*_ follow the triangle-like phase matching condition in the *x* ~ *z* plane, with determined angles ${\theta }_{p}^{G}$ and ${\theta }_{F}^{G}$ versus the fixed *k*_*s*_ (in the *z-*direction). It is a typical phase-matching triangle, perfectly described in scenarios where all the participants are plane waves in shape for the degenerate FWM process. This leads to an almost sole emitting direction *θ*_*F*_ for the FWM beam, limiting the potential applications. Therefore, to find more possibilities in emitting directions, in a 3D space, without bringing in extra momentums, we shall “rotate” such phase matching triangle with the signal beam *k*_*s*_ acting as the rotating axis. This operation will certainly give rise to multiple emitting directions (equivalently, multiple nonlinear refractions) for the controlled FWM beams. Then, the phase-matched wavevectors projected in the y ~ z plane (Fig. 1b), marked as 2*k*_*py*_ and *k*_*Fy*_ form a new triangle-like relation. Thus, a new pair of angular relations with ${\theta }_{py}^{G}$ and ${\theta }_{Fy}^{G}$ is established. Another version in plane *x* ~ *y* of Fig. 1c helps us understand more about the participants’ relations. The aforementioned wavevectors projected in the *x* ~ *y* plane are denoted as *k*_*pϕ*_ and *k*_*Fϕ*_, maintaining collinear. There emerges an important parameter, angle *ϕ*, quantifying the degree where the “rotation” behavior of the pump beam has reached. All the possible endpoints for *2k*_*pϕ*_ and *k*_*Fϕ*_ lead to a dashed circle in the *x–y* plane. We can find that the controlling pump beam and the controlled FWM beam will share a series of continuous one–one correspondent refraction relations (*θ*_*Py*_* vs. θ*_*Fy*_) in the version of Fig. 1b (*y* ~ *z* plane), determined by angle *ϕ*. Such induced nonlinear refraction relations are quantified in form of Eqs. (5) and (6), not fixed anymore.

5 $$2k_{py} \sin \theta_{py}^{G} = k_{_{Fy}} \sin \theta_{Fy}^{G} .$$

The maximum angles for *θ*_*Py*_ and *θ*_*Fy*_ are *θ*_*P*_*(6.44°)* and *θ*_*F*_*(9.34°)*, where *ϕ* is at 0° or 180°. Equation (5) can be expressed in form of *θ*_*py*_ and *θ*_*Fy*_, thus angular relation outside the glass:

6 $$n(\phi ) = \frac{{\sin \theta_{Fy} }}{{\sin \theta_{py} }} = \frac{{2k_{p} }}{{k_{_F} }} \cdot \frac{{\sqrt {\left( {{1 \mathord{\left/ {\vphantom {1 {n_{p} }}} \right. \kern-0pt} {n_{p} }}} \right)^{2} - \sin^{2} \theta_{p}^{G} \sin^{2} \phi } }}{{\sqrt {\left( {{1 \mathord{\left/ {\vphantom {1 {n_{_F} }}} \right. \kern-0pt} {n_{_F} }}} \right)^{2} - \sin^{2} \theta_{F}^{G} \sin^{2} \phi } }} .$$

In the above equation, the sole variant is *ϕ* from the *x–y* plane, not only presenting a new degree of freedom in controlling such nonlinear refractions but also implying an equivalent phase variation that distributes transversely along the flat glass slab(modulated by the effective refractive index *n(ϕ)*) as a whole, leading to spherical focusing of FWM beam. We can find that when *ϕ* ideally varies in the maximum range of 0–180°, it exhibits a complete high-order polynomial profile. To quantify, the maximum variation of such (effective) nonlinear refractive index from about 1.445 to 1.44935. The value 1.445 stands for the situation at the very edge of the focusing GRIN lens-like flat slab in full scale, while the value 1.44935 represents the central part of such a flat slab. When we concentrate on the central region around 90°, at paraxial condition, as shown in Fig. 1c, there present the nonlinear refractions on the edges of the scenarios in Fig. 1d and e, highlighted by dashed shadowed quadrangles. Such profile in phase variation resembles a typical type of GRIN lens parabolically featuring a spherical defocusing (Fig. 1d) or focusing (Fig. 1e) depending on the input pump beam profiles. This leads to a nonlinear tunable flat slab controlled by the wavefront shaping of the pump. Spherical wavefronts of the pump beam will satisfy many of the phase matching conditions under such “rotation” behavior, generating a more bent spherical wavefront. Again in Fig. 1d and e, the discrete arrows pointing to different directions in blue indicate a series of spherical wavefronts of the pump.

## Results

### Nonlinear refractions via FWM

**Figure 2:**
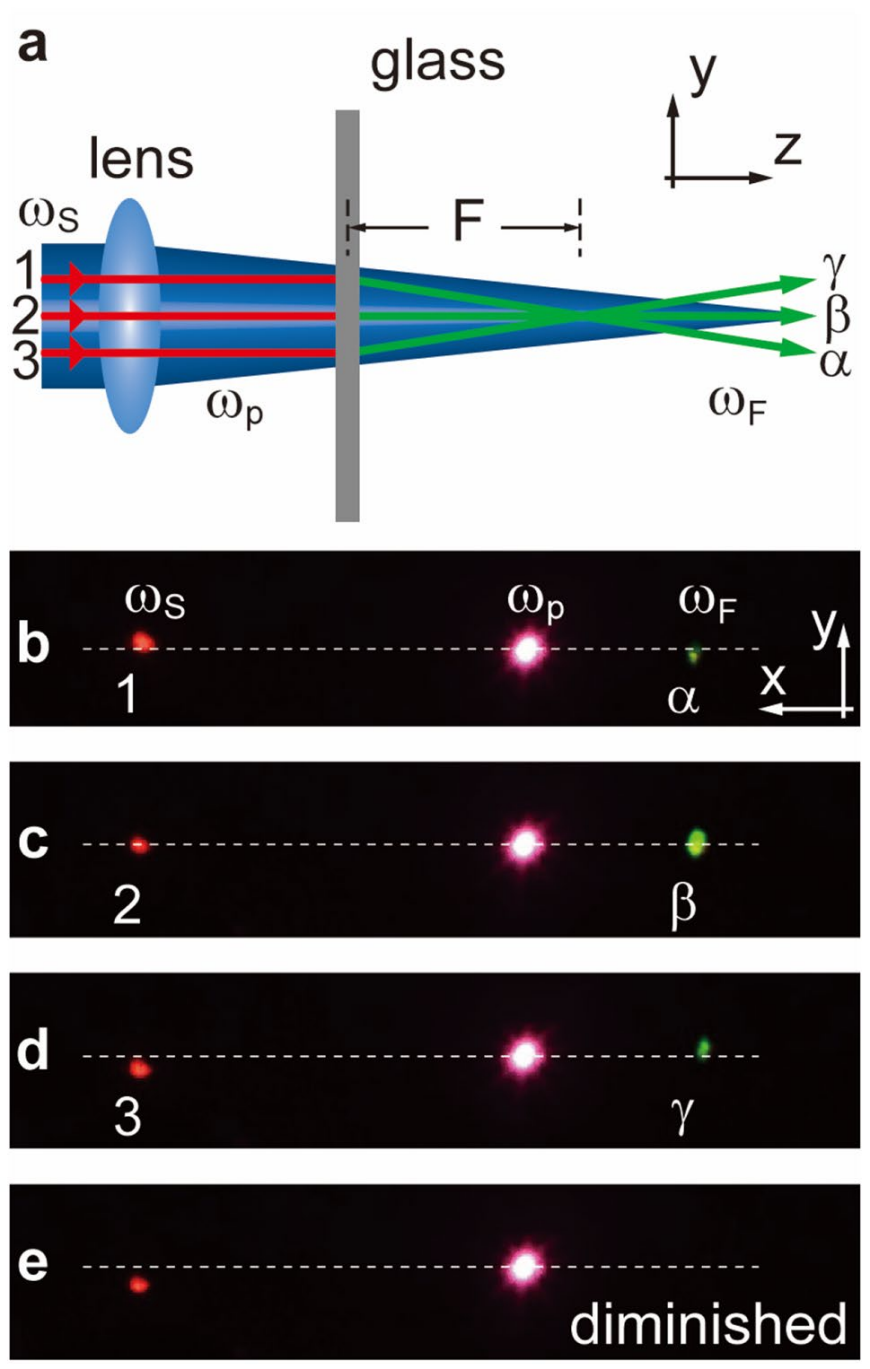
Nonlinear refractions across a focusing pump. By properly positioning the signal across different converging regimes of the pump in the flat slab, several nonlinear refraction relations of varying *ϕ* are shown in (**b**–**e**). *F* denotes the effective focal length for refracted FWM beam. (**a**) Experimental setup and analysis of FWM beams’ trend in the *y* ~ *z* plane. (**b**–**e**) Images on the target screen (*x–y* plane) in the far field. Leftmost red spots: signal 1300 nm, centered red: pump 800 nm, rightmost green spots: FWM beam 578 nm. The horizontal dashed lines serve as references. The FWM spot is upshifted (**α** to **γ**) when moving the signal spot downwards from **1** to **3**. However, due to the limited phase-matching range, the FWM disappears in (**e**).

To verify the above theory, firstly we experimentally examine the nonlinear refraction of the signal through wavefront-shaping of the pump beam in form of point-point correspondence, at some discrete points over the entire pump’s profile. According to the aforementioned theory, such nonlinear refraction manifests itself when the FWM beam is refracted in the *y* ~ *z* plane (Fig. 2a) depending on the incident angles of the pump, while the signal remains normal a plane wave to the slab. All the images are taken on a screen (a photosensitive card) of the *x* ~ *y* plane in the far field. It results in red spots in the middle as a reference. The 1300 nm IR signal on the leftmost is converted via FWM to the visible spots at 578 nm on the right (green). As shown in Fig. 2b–d, the pump is purposely focused through a lens to create a converging wavefront inside the glass slab, such that the FWM is indeed tilted when the incident signal sits on the different spots: when the signal moves downwards from spot **1** to **3** (Fig. 2b–d), the FWM spots (green, rightmost) arise in the opposite upward direction (**α** to **γ**). However, due to the limited spatial frequencies of *k*_*p*_,* ϕ* provided by the focusing pump which only varies in a range of 90 ± 3.83° in the current setup, making the FWM spot diminish in Fig. 2e. The convergence angle for modulated FWM beam(*θ*_*Fy*_) here is only about 0.63°. This result may not be so obvious, because the convergence angle for the focusing pump(${\theta }_{py}\approx {\theta }_{p}\cdot \mathit{cos}\phi$, paraxial approximation) is not significantly large either. Therefore the FWM beam could only exist within the tolerance near the exact phase matching condition, similar to the prior works^33,35^.

**Figure 3:**
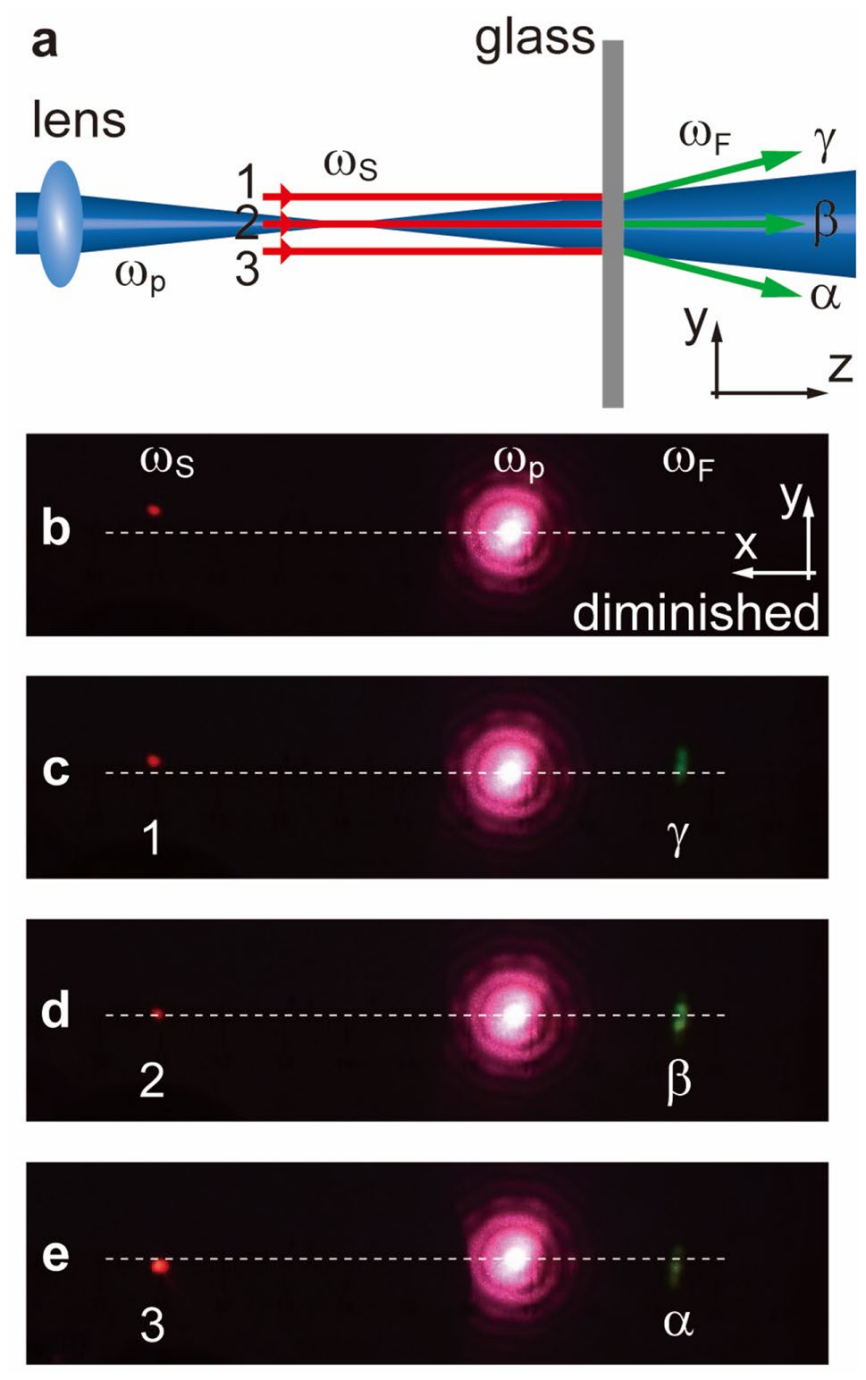
Nonlinear refractions across a defocusing pump. By properly positioning the signal across different diverging regimes of the pump in the flat slab, several nonlinear refraction relations of varying *ϕ* are shown in (**b**–**e**). (**a**) Experimental setup: the generation of the FWM beam in the *y* ~ *z* plane is predicted to be diverging. (**b**–**e**) Images on the target screen (*x–y* plane) in the far field. Leftmost red spots: signal 1300 nm, centered red: pump 800 nm, rightmost green spots: FWM beam 578 nm. The horizontal dashed lines serve as references. The FWM spot is downshifted (**γ** to **α**) when moving the signal spot downwards from **1** to **3**. However, due to the limited phase-matching range, the FWM disappears in (**b**).

Similarly, we show an opposite trend of nonlinear refraction can occur with a diverging pump. Instead of the converging pump, the slab is moved to the diverging regime after the pump’s focus (Fig. 3a). In this case, when moving the signal downwards again, the corresponding FWM spot now shifts downwards from **γ** to **α** as shown in Fig. 3b–e, this is exactly opposite to the former case with the converging pump. Here the actual range of *ϕ* is not large either, only in an efficient range of 90 ± 1.28°, making the *θ*_*Fy*_ up to only 0.21°. Such divergent angle range seems to reduce more compared to the former case, this is because a comparatively shorter focus(~ 10 cm) for the pump here as compared to ~ 25 cm’s focus in the former case induces more phase mismatch in FWM process, limiting FWM’s angle. Here, it’s better to use one uniform lens for the same convergence or divergence. However, the purpose of this section only aims at consistent or opposite moving trends for controlled FWM beam versus pump, which has primally demonstrated that the spherically shaped pump controls the FWM beam’s wavefront with a sketch of point-point correspondence, at some discrete points.

### Nonlinear flat lens

**Figure 4:**
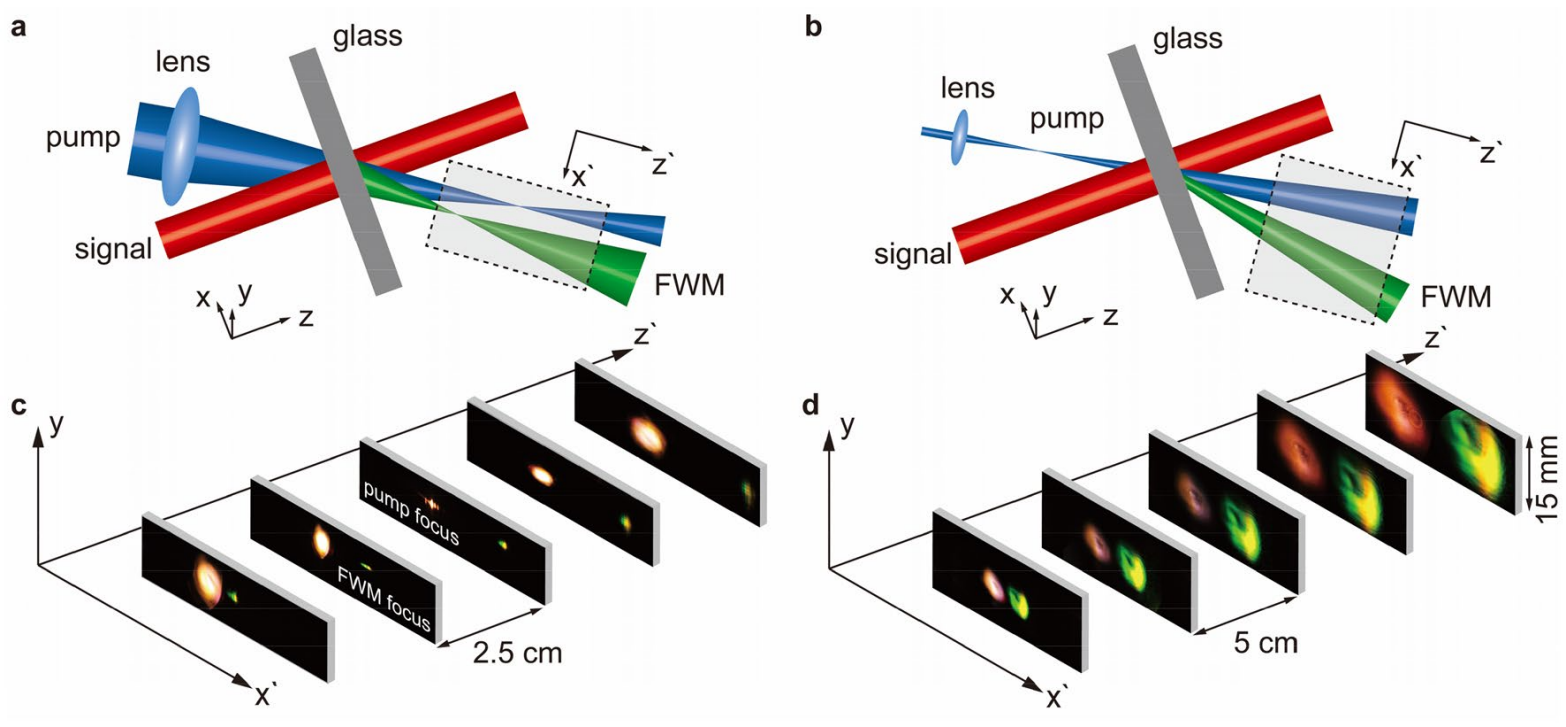
Beam evolution through a nonlinear flat slab in the transverse plane. (**a,b**) show the experimental schematics of a normal incident signal onto the flat slab with a converging (**a**) and a diverging (**b**) pump beam. A series of images (**c**,**d**) are taken within the shadowed area behind the flat slab. (**c**) A scan with a 2.5 cm increment from 10 to 20 cm after the glass slab along the path z` unveils the relevant beams’ transverse (*x`* ~ *y* plane) evolution trends for the nonlinear flat lens scheme, showing a focusing FWM beam. Its focus is observed at around 12.5 cm when the pump’s focal point is placed at around 15 cm from the slab. In a contrast, (**d**) a similar scan reveals a diverging nature of the FWM beam behind the flat slab when the pump beam is diverging as well. The furthest FWM beam spot reaches a diameter of up to 15 mm at a distance of 27.5 cm, the nonuniform wavefront of the FWM beam may be caused by the nonuniform pump beam.

In Fig. 4, to fully demonstrate that the pump’s wavefront can reshape the nonlinear FWM beam, we perform a series of FWM experiments as well as numerical simulations (see supplementary information) through the flat slab to construct effective focusing/defocusing FWM lenses by implementing a wavefront-shaped pump onto the slab. Still the signal is kept plane wave. Figure 4a shows the experimental setup of the FWM lens: by focusing the pump onto the slab, the converging wavefront of the pump can steer the FWM beam, also in a converging manner, effectively forming a GRIN-like lens. Both the conventional GRIN lens and our nonlinear flat slab here can reshape the wavefront not relying on traditional geometric curvatures but the indexes(phase) variations underlying beneath the flat uniform appearances. As a result, the FWM beam is also focused, the focal length for the FWM beam is closer to the slab in comparison to the one for the pump. This phenomenon attributes to the phase-matching relation in Fig. 1 that *θ*_*F*_ is slightly larger than *θ*_*P*_. Note that this feature of a nonlinear flat lens can be controllable and compact with a better focusing performance. By controlling the distance from the flat slab to the focal point of the pump beam under phase matching conditions, the focal length for the controlled FWM beam can be tuned continuously within the maximum convergence angle *θ*_*F*_. In another word, utilizing Abbe Theory, the theoretical resolution limit of *0.61λ/NA* could be improved from 4.35 μm (pump, phase matching convergence *θ*_*P*_) to 2.17 μm (FWM beam, maximum convergence *θ*_*F*_) for future imaging applications^33,36^.

Figure 4c lists an array of images lining along the direction z` from the shadowed area of the setup in Fig. 4a. Images are taken from 10 to 20 cm after the glass slab along the path z` in a 2.5 cm increment. We use a camera with only a viewfinder (size: 23.6 × 15.8 mm) to capture the images in the *x`* ~ *y* plane (transverse plane). Since the viewfinder’s size is adequate for most situations, we can directly collect the wavefronts for either the pump or the FWM beam’s evolution trends. The red spots denote the pump and the green-yellow ones for the FWM beams. The results clearly show that with a converging pump’s wavefront, we can realize the generated FWM in a converging wavefront too. Differently, the focal length for the FWM beam is indeed shorter than the distance of the focal point to the slab for the pump, which agrees well with our analysis above. The lens modulating the pump is ~ 25 cm focus. We place the slab 15 cm before the pump’s focus while it is 12.5 cm for the modulated FWM to the slab, which proves an effective flat lens operating on FWM frequency with better convergence. The collected beam spots are converging well in the *y* direction, this is the result of our previous work in^33,35^, indicating an anisotropy in non-collinear phase matching.

We can realize an effective nonlinear defocusing flat lens in a similar way. The lens modulating the pump is still ~ 25 cm focus. Instead, we implement a defocusing pump onto the dielectric slab. In Fig. 4b, by a proper arrangement of the lens, the pump shall focus at a distance of 14 cm before the slab, creating a defocusing trend in the slab. As shown in Fig. 4d, the FWM beam exhibits a divergent trend following the divergent pump beam. Images are taken from 7.5 cm to 27.5 cm after the glass slab along the light path *z`* in a 5 cm increment. The results prove that a diverging wavefront of the pump can reshape the generated FWM in a similar profile with a focal length for FWM shorter than that for the pump, which presents a bigger size for the FWM spot in each image. Due to the laser pulse bandwidth and glass thickness-induced tolerance, we can observe such multicolor FWM beam profiles, similar effects have also been reported previously^33^. Note that the furthest image for the FWM beam spot evolves too large to fully maintain in our optical system, thus the image appears intercepted by a partially circular-like border. The related *ϕ* in this case turns out to be broadened in a range of 90 ± 9.5°, and the related divergence angle for modulated FWM here is also enlarged to 1.55°, indicating a variation of about 1.2 × 10^−4^ in effective nonlinear refractive index from Eq. (6). It’s also interesting to find that the images for FWM beam spots in Fig. 4d are hollow-core-like. Since we do not intend to operate on the laser’s original Gaussian output, the reason partially attributes to the pump’s central area with high intensity that partly joins the birth of the signal in OPA, thus losing part of the energy needed to generate FWM beam, making a similar hollow-like transverse profile. And mainly due to the deteriorated phase mismatching in the *x* direction that cannot generate a significant FWM beam.

### FWM beam steering

**Figure 5:**
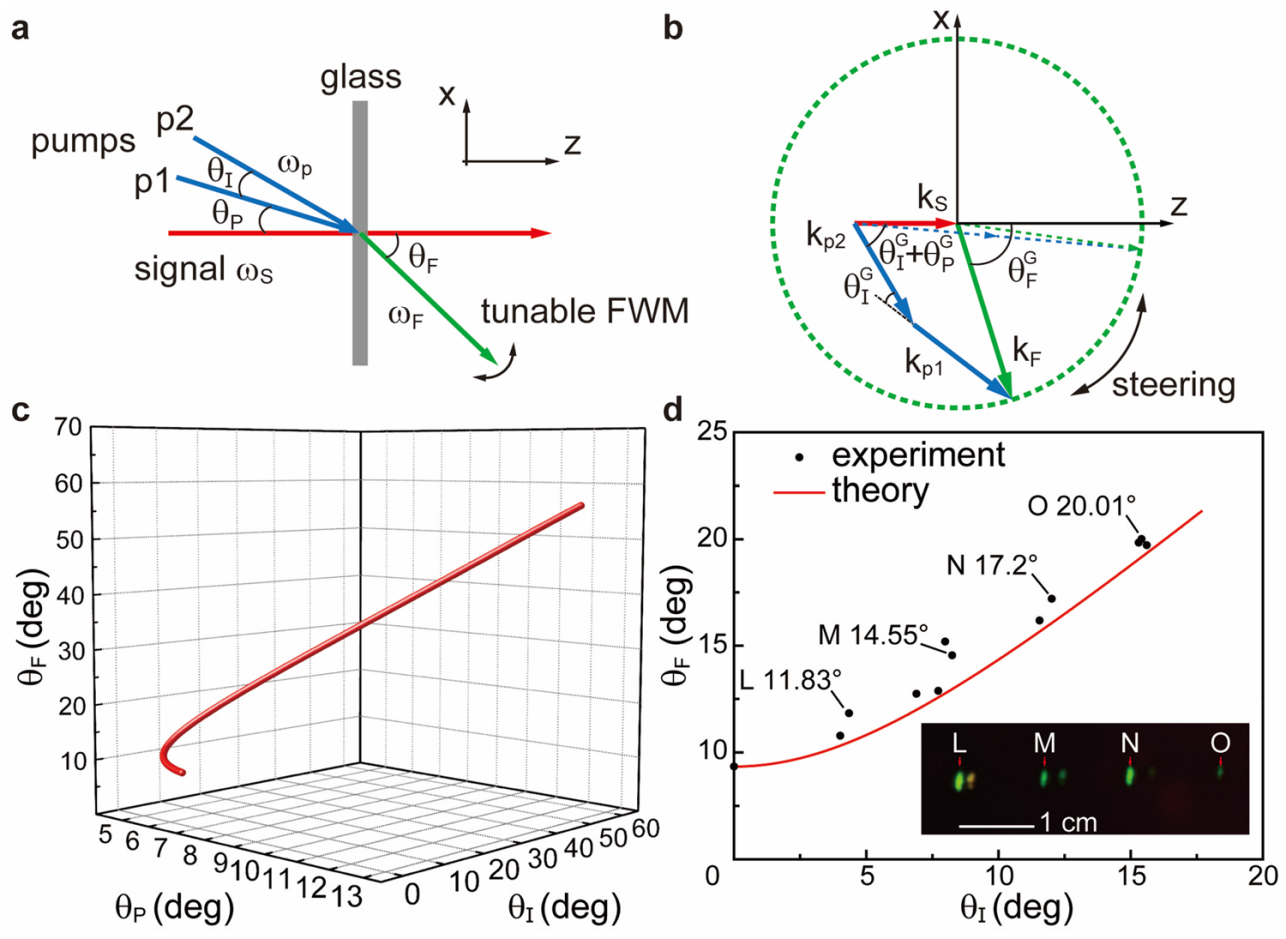
Demonstration of nonlinear FWM steering to a large angle. **(a**) In contrast to the degenerate one, the non-degenerate pumps now are separated with *θ*_*I*_ denoted as the intersection angle between p1 and p2, *θ*_*p*_ as p1 to the signal(normal incidence). By properly choosing these angle relations and considering the phase matching, the FWM beam can be steered to a wider angle *θ*_*F*_ as shown in (**b**). In this manner, the FWM beam can be steered from 9.34° to nearly 90° (6.14°—41.23° for ${\theta }_{F}^{G}$) in the *x* ~ *z* plane. The dashed green circle implies all possible endpoints for tuned FWM beam vectors within the glass slab. (**c**) All the possible steering angles in a 3D angle box with two input variants, i.e., *θ*_*p*_ & *θ*_*I*_. (**d**) Experimental measurements for the non-degenerate FWM steering angles based on (**b**). We define the variant *x*-axis as the intersection angle for the two pumps(*θ*_*I*_). Black dots are experimental results and the red curve presents the theoretical calculation from Eq. (9). The insert slice denotes the images taken 210 mm away from the glass slab demonstrating a beam steering at angles 20.01º(**O**), 17.20°(**N**), 14.55°(**M**), 11.83°**(L**).

In the previous section “Nonlinear refractions via FWM”, we have proposed a controllable method to realize a series of nonlinear refractions by certain wavefront profiles of the pump. Further by proper modulations, the generated FWM beam tends to focus or defocus, making an effective flat lens. However, the angle tolerance or tunability is limited due to phase mismatching. Even if we made the *ϕ* varies to the maximum range, the emitting angles of the FWM beam for the above methods are still limited by the maximum *θ*_*F*_*(9.34°)* in the version of the *y* ~ *z* plane. For a larger angle steering application, we can consider a non-degenerate FWM scheme in plane waveform. For instance, the non-degenerate FWM scheme, obeying phase matching relation in Fig. 5a and b, has divided the ever-collinear pump wavevectors into separate *k*_*p1*_ and *k*_*p2*_, respectively. The two are equal in absolute value but different in spatial direction(wavevectors), introducing one more variant *θ*_*I*_*.* Therefore, by a cautious arrangement of the pump wavevectors, we are able to seek the existence of FWM pointing at multiple angles. In comparison, the degenerate FWM process conducted in the nonlinear flat lens demos is a conventional one with the feature of a steady phase matching triangle which lacks flexibility. By making two vectors of the pump non-collinear we could acquire some flexibility in the nonlinear process, transforming the fixed phase matching triangle into a tunable phase matching quadrangle.

From Fig. 5b, the phase matching curve viewed from the *x* ~ *z* plane indicates the possible beam steering range by tunable FWM process within the glass slab. The phase matching fulfills $\vec{k}_{p1} \, + \,\vec{k}_{p2} \, - \,\vec{k}_{s} \, - \,\vec{k}_{_F} \, = \,0$, still with the signal normal incident. The dashed circle in green intercepted by *k*_*F*_(green arrow) suggests the directions of all the possible endpoints of the generated FWM wave, estimating steering ranging from about 9.34º to nearly 90º for *θ*_*F*_ (6.14–41.23° for ${\theta }_{F}^{G}$). The phase matching triangle in dashed arrows stands for its minimum steering angle while two pumps are collinearly incident, the same as the previous degenerated one. In the presented non-degenerate case, such phase-matching quadrangles can reach the maximum steering condition theoretically by a cautious arrangement on ${\theta }_{I}^{G}$ and ${\theta }_{P}^{G}$, leading to a tunable FWM steering to large angles. The actual phase matching condition in this case now changes to (also in *z* and *x*):

7 $$k_{s} = k_{p} \left[ {\cos \theta_{p}^{G} + \cos \left( {\theta_{p}^{G} + \theta_{I}^{G} } \right)} \right] - k_{_F} \cos \theta_{F}^{G} .$$

8 $$k_{p} \left[ {\sin \theta_{p}^{G} + \sin \left( {\theta_{p}^{G} + \theta_{I}^{G} } \right)} \right]\, = \,k_{_F} \sin \theta_{F}^{G} .$$

Similarly, we could combine linear Snell’s Law of refraction to Eq. (8), deducing a nonlinear FWM Snell’s Law for such a non-degenerate case:

9 $$\frac{{\sin \theta_{F} }}{{\sin \theta_{p} + \sin \left( {\theta_{I} + \theta_{p} } \right)}} = \frac{{\lambda_{F} }}{{\lambda_{p} }}$$

Figure 5c theoretically unveils the multiple relations of the FWM parameters mentioned as *θ*_*p*_*, θ*_*I*_ and the steering angle *θ*_*F*_ in a 3D figure form based on Eq. (7) and (9). It indicates a clear monotonic relation for *θ*_*I*_ and *θ*_*F*_. Anyway, it becomes partially non-monotonic for *θ*_*p*_ versus the other two angles. Around the value of *6°* of *θ*_*p*_, in coincidence with our experiment results, there will be much more tolerance for FWM generation since one value for *θ*_*p*_ ranging from *4.82°* to *6.45°* can lead to two FWM steering angles *θ*_*F*_ according to curves in Fig. 5c. It is consistent with the insert image of Fig. 5d, presenting multiple-angle steering for FWM beams at the same time. While *θ*_*p*_ keeps increasing, the angle relations will turn monotonic again.

In Fig. 5d, the experiment is carried out still using the same BK-7 glass slab of 1 mm thickness and results depict a beam steering angle relationship between *θ*_*I*_ (two pumps’ intersection angle) and emitted FWM, *θ*_*F*_. They display a monotonic dependence which may reduce some complexity in conducting the setup. The black dots are the ones measured in the experiment and the red cure is the computational simulation according to multiple angles’ phase matching conditions in Eqs. (7), (8) and (9). An extra degree of freedom is induced by the newly participant wavevector *k*_*p2*_. And the full scales of each beam spot(especially pumps’) during the measurement will also enlarge the FWM beam spot size. Despite some difficulties in constructing the setup, we manage to demonstrate an FWM beam steering from its minimum (9.34º) to over 20º, while the intersection for pumps varies from 0º to about 15º, making the signifying factor^37^ for steering less than 2. It is a born defect that the wavevectors *k*_*F*_ and *k*_*p1,2*_ for the FWM beam and pumps are of the same order of magnitude, thus the signifying factor value will not be so large as tens to hundreds. In a nonlinear DFG process of THz generation and steering^38^, the factor could reach a very high value due to the huge difference for wavevectors of optical and THz. The image slice inserted in Fig. 5d is captured under the matching conditions within the tolerance that four different steering angles(beam spots) all show up for once. In practice, we use an optical transmitting grating to produce multiple interception angles *θ*_*I*_ for the pump via different orders of refractions. The insert image is taken 210 mm away from the glass slab indicating a beam steering at 20.01º, 17.20º, 14.55º and 11.83º against normal incidence. These results can also be verified by numerical simulations in the supplementary information.

## Conclusion

We have demonstrated a fabrication-free tunable flat slab using nonlinear a four-wave mixing process by wavefront-shaped pump beams. This technique can provide certain wavefront manipulations through nonlinear wave mixing without the need of fabrication. To beam steering applications, our setup could reach ~ 20°, far larger than a degenerate case which is around 1° in Ref.^33^. and slightly larger than a similar DFG scheme which is about 17° in Ref.^35^; Moreover, the nonlinear FWM process also enables up-conversion imaging, which has found its applications in nonlinear superresolution^33,36^. Furthermore, the nonlinear phase matching process occurs inside the bulk slab, not along the 2D metasurfaces, this extra dimension combined with a spatial light modulator, may allow more functional wavefront shaping in the near future. Such flat slab unveils a new way of pure light manipulation for wavefront control and shaping process, showing considerable potential in adaptive imaging and all-optical information applications.

## Supplementary Information

Supplementary Information.

## Data Availability

The datasets analyzed during the current study available from the corresponding author on reasonable request.
