# Supplementary material for: Wavefront shaping with nonlinear four-wave mixing
Source: Sci Rep. 2023 Feb 16;13:2750. doi: 10.1038/s41598-023-29621-w (PMC9935867; doi:10.1038/s41598-023-29621-w)
Supplement: Supplementary file 1 — Supplementary Information. [file 41598_2023_29621_MOESM1_ESM.docx]

Supplementary information for

**Wavefront Shaping with Nonlinear Four-wave Mixing**

**Dongyi Shen^1^, Jianjun Cao^2^, and Wenjie Wan^1,3*^**

^1^State Key Laboratory of Advanced Optical Communication Systems and Networks,

School of Physics and Astronomy, Shanghai Jiao Tong University, Shanghai 200240, China

^2^School of Science, Jiangnan University, Wuxi 214122, China

^3^University of Michigan-Shanghai Jiao Tong University Joint Institute, Shanghai Jiao Tong University, Shanghai 200240, China

*Corresponding authors: Wenjie Wan [wenjie.wan@sjtu.edu.cn](mailto:wenjie.wan@sjtu.edu.cn)

**S1. FDTD simulations for verifying wavefront manipulation methods proposed in experiments**

we have performed several additional numerical simulations based on finite difference time domain (FDTD) method. These simulations are meant to demonstrate most of what we have shown in the experiments: 1. The existence of the degenerate FWM beam in plane wave profile. 2. The unique wavefronts features of the controlled FWM beam when the controlling pump is in spherical profiles. 3. Wide-angle FWM beam steering in a non-degenerate form.

In these simulations (Fig. S1-Fig. S5), the signal beam is set to be normally incident(towards *-z* direction) into a BK-7 flat slab(thickness 20*μm*) with a plane wave source of a bigger scale. The controlling pump beam is launched from a Gaussian source of a smaller scale. In Fig. S1b, when the pump beam divergence(“*NA*”) is set to be very small, it can indeed be treated as a plane wave within the observed area. The incident angle for the pump beam is set to be *θ_P_(6.44°)* according to the phase matching condition. The FWM beam with a plane wave profile is generated accordingly in Fig. S1c. The electric field distribution shows a very good agreement with the theoretical calculations.


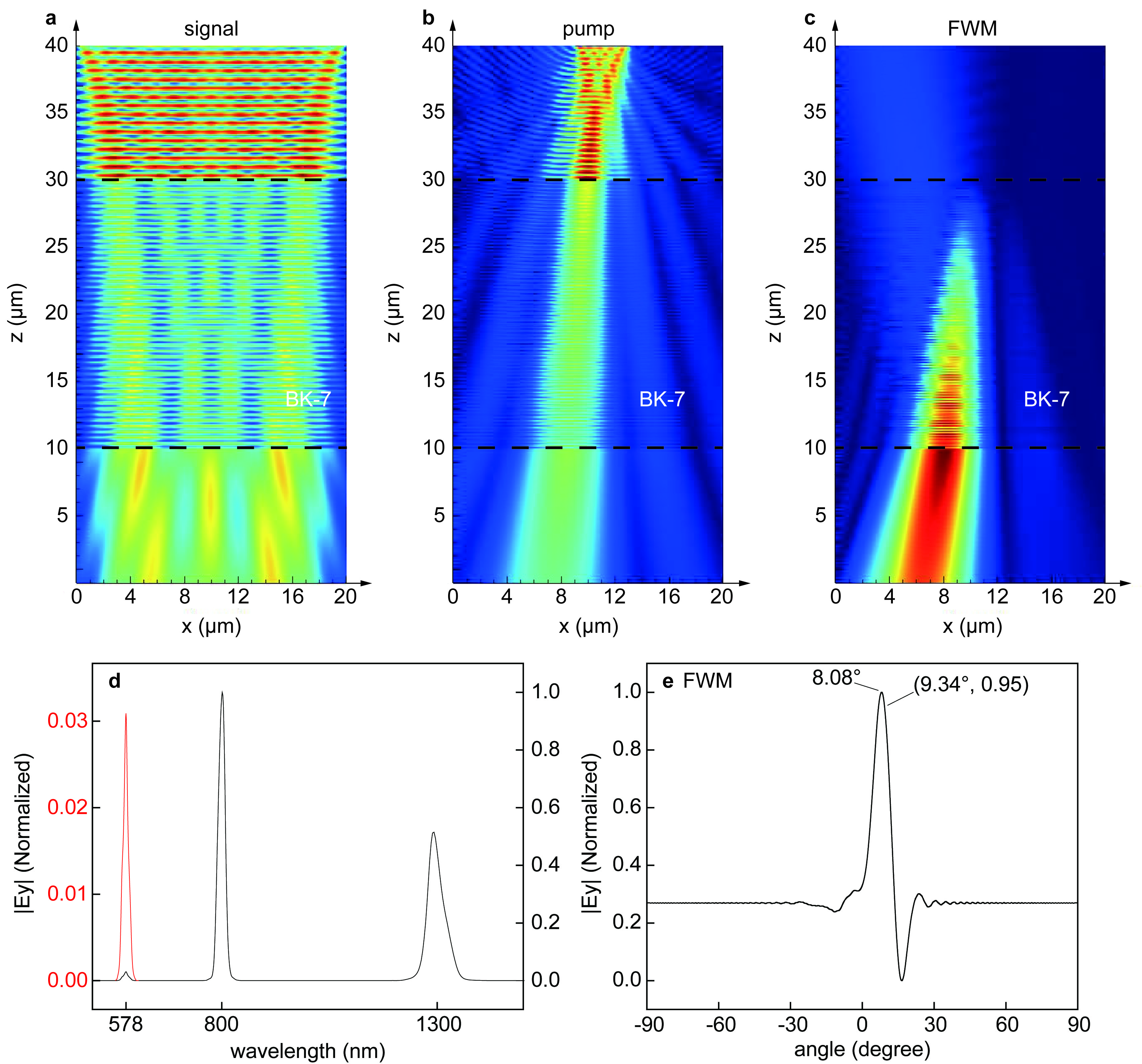


**Fig. S1 FDTD simulation for FWM generation in plane wave profile.**

Fig. S1d shows a relative electric field comparison of the FWM process participants in the spectra region. It is measured through a field-time monitor placed in the middle of the slab. Since the simulations are operated in TE polarization, *|Ey|* acts as the measurement of the electric field. In addition, a line monitor can calculate the far-field emitting angles for the desired FWM beam in Fig. S1e. The peak sits at an angle of 8.08°, with a small error to our calculated and experimental data of 9.34°(in main text Figure 2 and 3 for *x*-axis). It is tolerable considering a minor drop from 1 to 0.95 in normalized *|Ey|*.


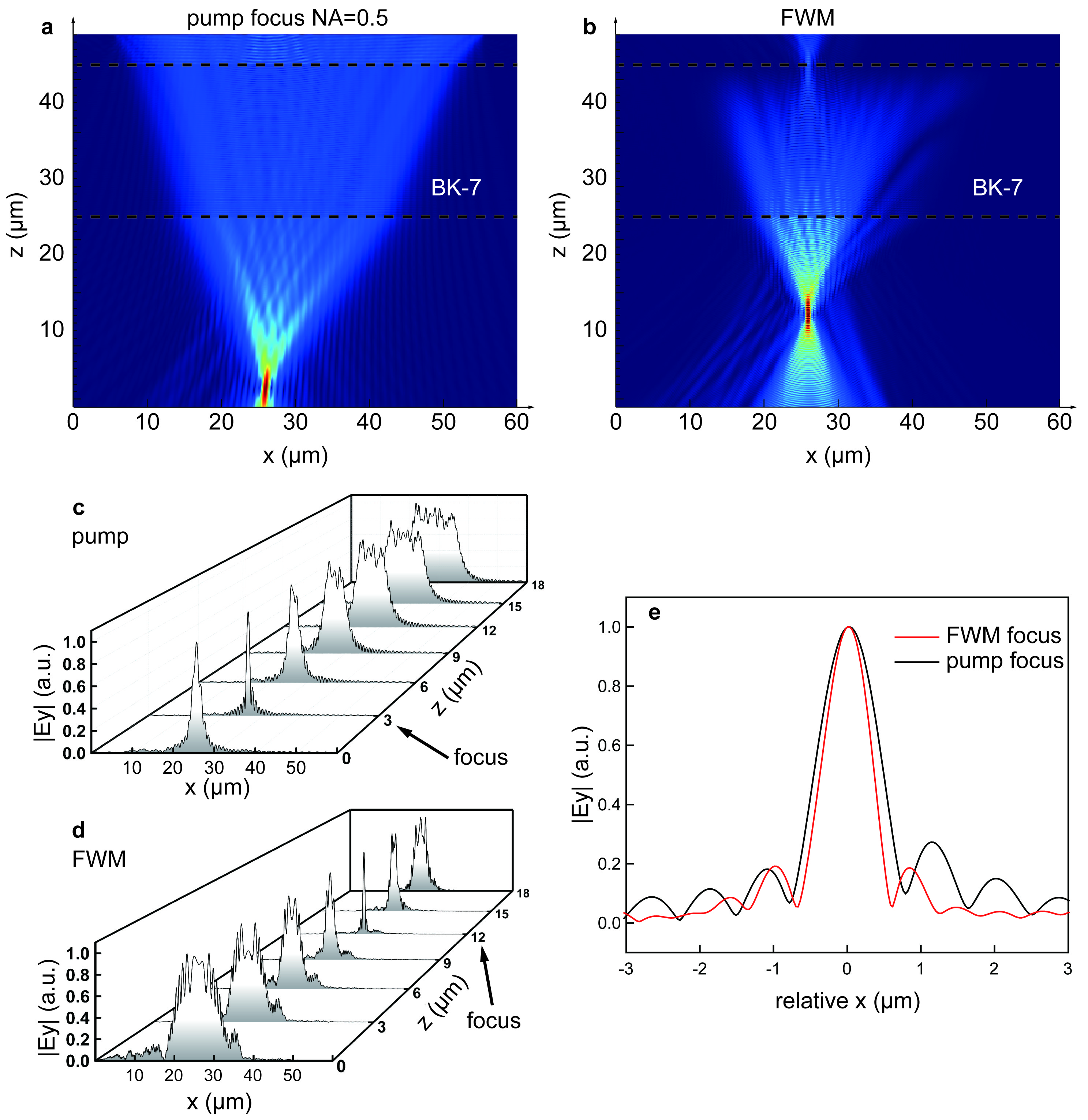


**Fig. S2 FDTD simulation with controlling pump in a spherical focusing trend of *NA=0.5*.**

Fig. S2 and Fig. S3 are our simulations for demonstrating a series of spherical focusing FWM beams. By making the controlling pump beam in a spherical profile respectively with *NA=0.5* (Fig. S2) and *NA=0.3* (Fig. S3), we see much of the agreement to our experiments in main text Figure 4a and 4c. Such unique feature lies in a more bent (closer focal point to the slab) compared with the controlling pump due to phase matching. Fig. S2c and S2d are the lists for spatial evolutions of the beams’ transverse profiles(wavefronts), resembling our experiment results in the main text Figure 4c. Furthermore, the comparison between the two beams’ transverse profiles at their own focal points (Fig. S2e) also indicates a narrower FWM beam, that is, a better resolution in potential imaging. We have made such a theoretical prediction in the main text. However, what we have predicted is approximately 2 times the difference for 4.35*μm* (pump) to 2.17*μm* (FWM beam). Here only the FWM beam seems to match our prediction for the reason that such prediction of the pump beam’s profile is limited to the strict phase matching angle(6.44°, equivalently *NA*≈0.11), And such settings of a much smaller *NA* in this simulation environment will turn the results insignificant, making pump plane-wave like. To partially compensate for that, we continue to perform a simulation in a smaller *NA=0.3* while maintaining the pump’s focal point (Fig. S3a).


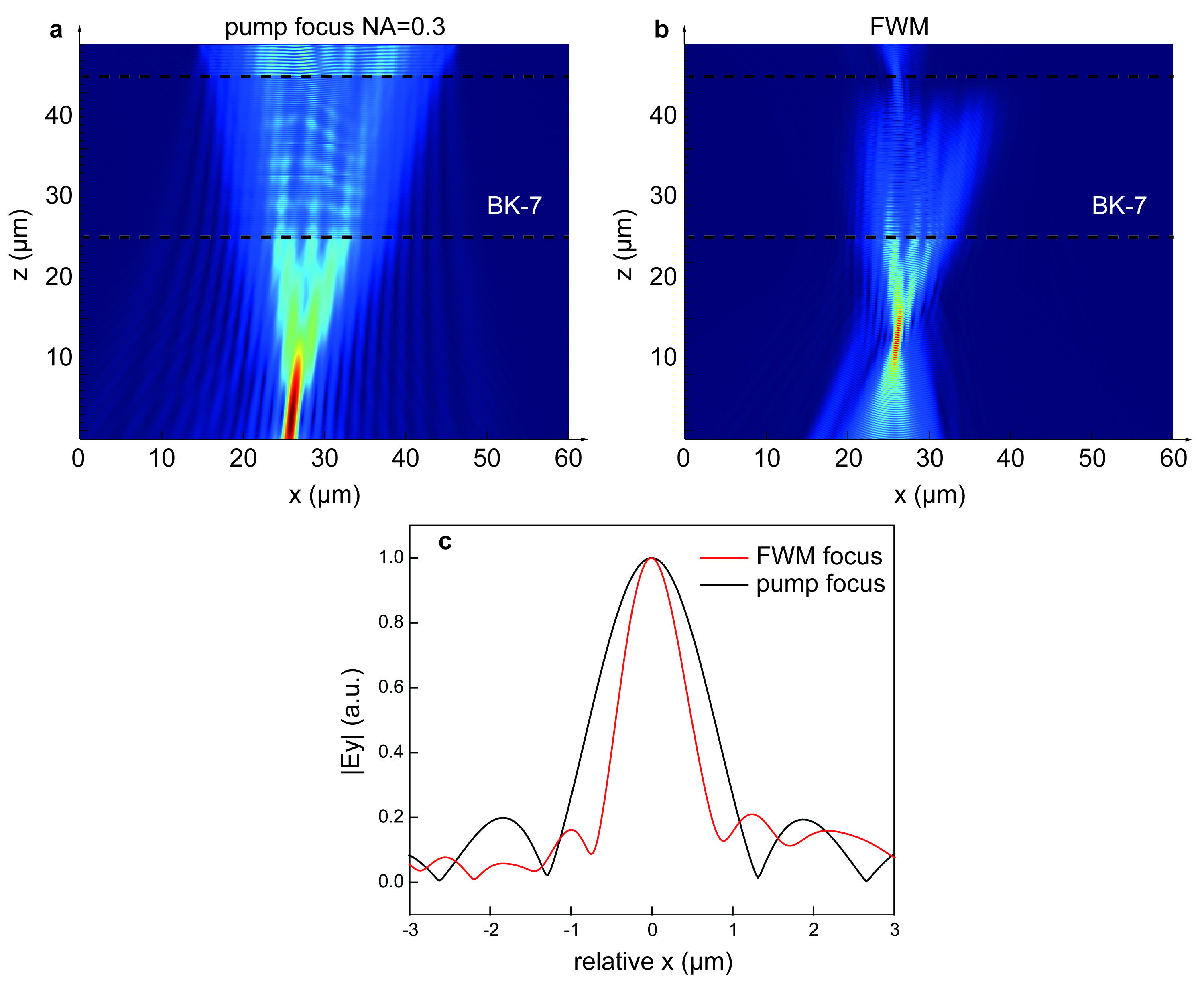


**Fig. S3 FDTD simulation with controlling pump in a spherical focusing trend of *NA*=0.3.**

In Fig. S3b and S3c, the location and the profile of the FWM beam at the focal point barely change compared with itself in Fig. S2b and S2e. This also attributes to a satisfied but limited phase matching. However, the pump’s profile at the focal point is enlarged to an observable extent compared with Fig. S2e. Such a trend for the pump beam in a smaller *NA* agrees with our prediction.


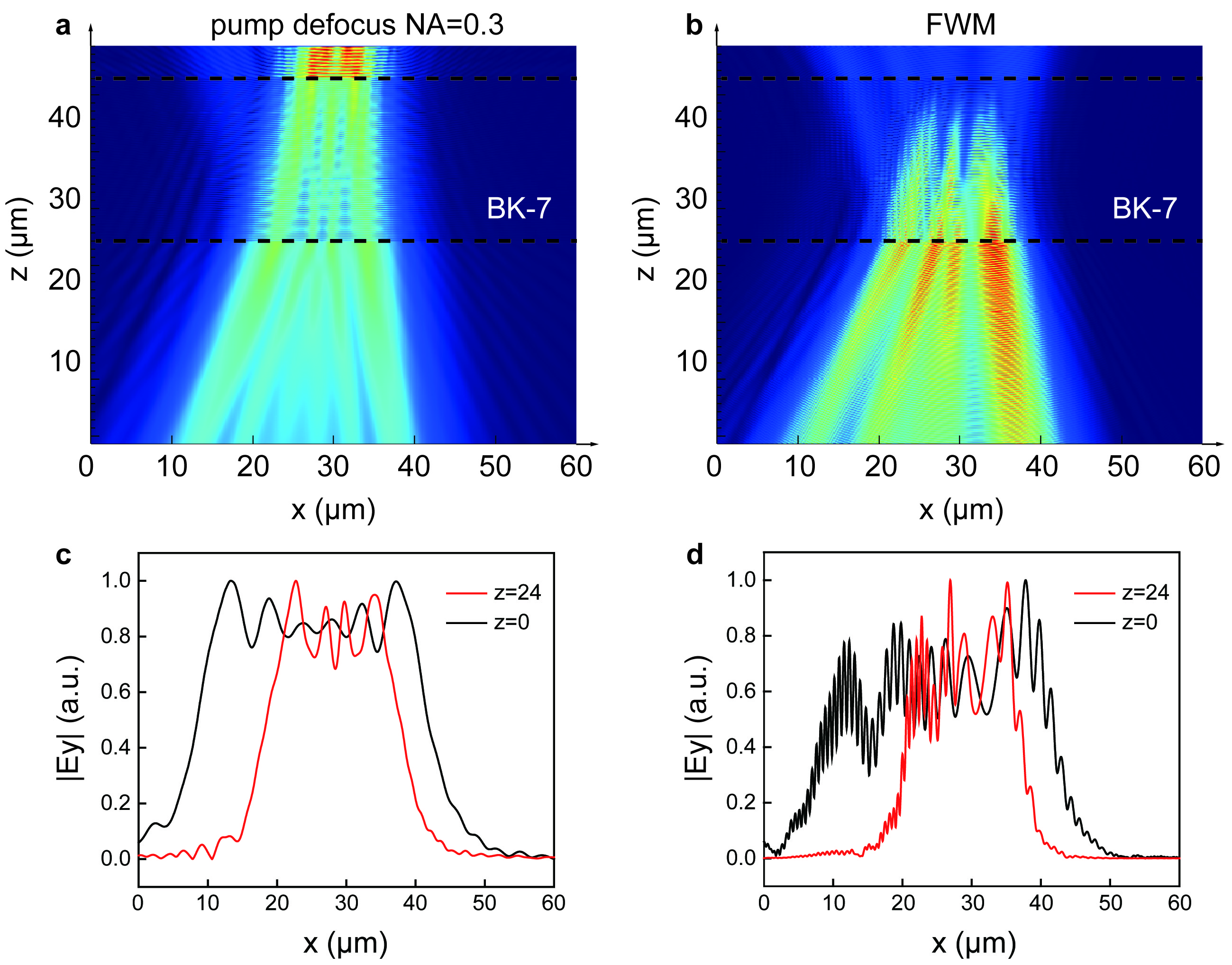


**Fig. S4 FDTD simulation with controlling pump in a spherical defocusing trend of *NA*=0.3.**

Next, Fig. S4 presents the simulation with a reversed, spherical defocusing trend of the pump beam with *NA*=0.3. The resultant, controlled FWM beam’s profile also diverges spherically, in accordance with our experiments in the main text Figure 4b and 4d. Such quantified trends of the simulation are shown in Fig. S4c and S4d. The defect remains that the pump beam’s *NA* is still beyond the strict phase-matching requirement, leading to an insignificant contrast in divergence angles for the two beams.


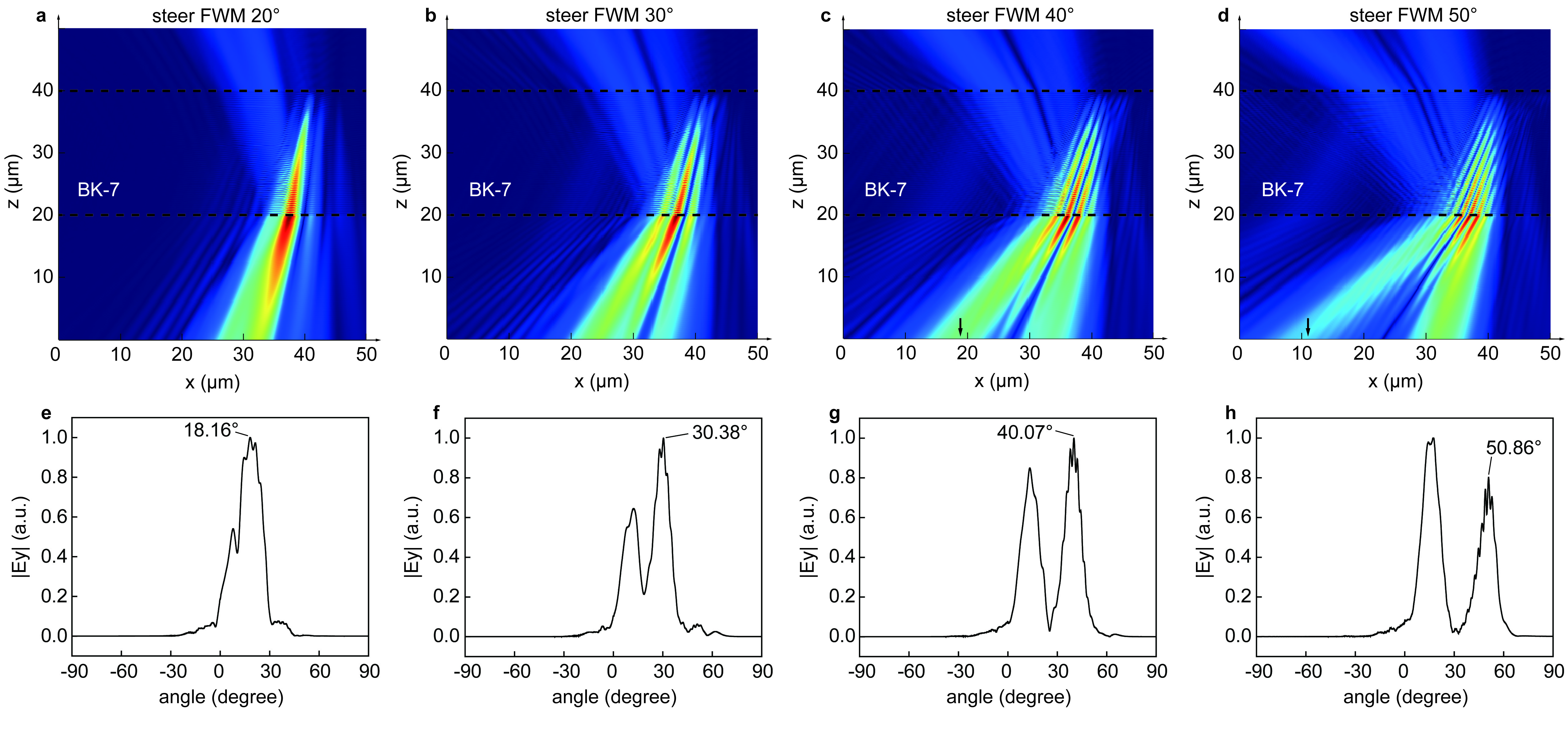


**Fig. S5 Wide-angle FWM beam steering in a non-degenerate plane-wave form.**

Finally, we intend to bend the FWM beam to a wide range of angles uniformly. The methods are non-degenerate FWM form, where we separate the pump beam into two independent controlling wavevectors and thus inject a new degree of controlling freedom herein. Fig. S5a-S5d are the electric field distributions of the controlled FWM beam steering based on the theoretical calculation in the main text. They are designed to be steered at 20°, 30°, 40°and 50°, respectively. The simulation results indicate the measured steered angles of FWM beams in the far field are at 18.16°, 30.38°, 40.07°and 50.86°, respectively (Fig. S5e-S5h). These are good verifications for FWM beam steering extended from our experiments in the main text Figure 5, except there stands another peak around 10° in every case. This attributes to the angles of pump1(*k1*), which vary in a smaller angle range, stimulating some degenerate FWM beam within the tolerance of the simulation. Another feature is the lowered electric field magnitude at larger angles(Fig. S5h). A lowered transmission rate into the slab due to the enlarged incident angles of pump2(*k2*) may explain this.

We should note that due to the limited performance of our software and the stability concern, the simulations are carried out in the *x-z* plane and output in one 2D slice. Instead, if we intend to operate the simulations in the *y~z* plane following the same beam vectors’ arrangement, it requires multiple slices to cover all the cross sections of the signals and pumps, much close to a 3D manner. That’s really challenging and we may do it in the future. Additionally, the thickness of the slab is set to be 20*μm*, which satisfies the temporal coherence concerns of femtosecond pulses, however, it is much shorter than our experimental parameter which is ~1*mm*. Surely, there will be more tolerance in FWM phase matching in simulations(less strict dependence on one or certain angle), which brings an extra momentum by $2\pi$/*(thickness)*. Comprehensively, we believe the above simulation results are capable of assisting the proposal of our experiments on most occasions.
